# Supplementary material for: Judgments of American English male talkers who are perceived to sound gay or straight: Which personal attributes are associated with each group of talkers?
Source: PLoS One. 2026 Apr 10;21(4):e0346897. doi: 10.1371/journal.pone.0346897 (PMC13068255; doi:10.1371/journal.pone.0346897)
Supplement: Attachment — Submitted filename: Review_PONE-D-25-51754.pdf [file pone.0346897.s001.pdf]

Review of Judgments of American English male talkers who are perceived to sound gay or straight: Which personal attributes are associated with each group of talkers?

This paper conducted a study that explored whether knowledge of a talker's sexual orientation would impact the perception of the talker sounding either "gay" or "straight" and whether any of the eight attributes (sad, confident, stuck-up, bored, outgoing, intelligent, mad and old) were associated with one group of talkers over the other. Stimuli consisted of 48 read tokens spoken by 24 males from Ohio (12 self-identified gay; 12 self-identified straight): each token concatenated monosyllabic CVC words that featured two instances of /s/, following previous work showing that listeners use /s/ to differentiate talkers. Stimuli were chosen based on listener judgments indicating that the talker was consistently perceived at one end of the gay-straight spectrum in a previous study conducted by the authors. Importantly, the authors acknowledged the gay-straight binary is not representative of the dynamic nature of sexual orientation, but they offer rationale to implementing the binary to measure for participants' judgments. The authors identified the study's distinct novel contribution of the manipulation of knowledge of talkers' sexual orientation. The sexual orientation label was varied between subjects. Three listener groups experienced one condition each: Not Referenced, Truthfully Referenced, or Falsely Referenced. For this study, participants were instructed to make a listening judgment using a 7-point Likert scale to indicate the strength of association of an attribute with sounding gay or straight. 210 participants from a rural southeastern university had been instructed to make judgments after each trial with the three different conditions. Results demonstrated that perceived-gay talkers were judged often as confident, outgoing, mad, and stuck-up, whereas perceived straight-talkers were judges more often as sad and old. Additionally, the labeled condition mattered for sad and stuck-up. These findings partially support their two aims with the critical findings that speech signal influenced listeners' judgments regardless of prior knowledge of the talkers' sexual orientation. Thus, the study advances research by directly contrasting self-identified vs. perceived sexual orientation and showing that perceived orientation drives key social judgments.

Regarding the methodology, the study introduces the manipulation talkers' orientation labels via truthfulness with listener judgments, an uncommon approach in the literature. The authors justified the use of a Likert scale to measure association between talkers and attributes mapped onto a listening judgment task. Thus, using mixed-effects Poisson regression is justified by the authors to be the more appropriate method in using the Likert scale. The authors acknowledge the implications of associating dynamic sexual orientation with a binary and appropriately state future research should focus on disentangling sexual orientation and binaries. The use of concatenated strings of monosyllabic CVC words is an appropriate stimulus for this study since the authors reasoned that content from spontaneous speech could influence judgments. A small note I have on the usage of "SO" for sexual orientation is the confusing instances where the full phrase is used. If "SO" is not a standard abbreviation, I recommend expanding the abbreviation for consistency as there are instances where "sexual orientation" is not abbreviated.

Regarding the listener sample, I have reservations about potential underlying influences on listeners' ements. The authors note that listeners were not probed for prior beliefs and qualitative details, but they acknowledge this as a limitation with future directions (citing Tripp & Munson, 2023). Additional emphasis, future experimental design suggestions or elucidation on why qualitative data was overlooked may enhance understanding given the central role social ideologies in work of this kind. This addition may relate to the listeners' "[desire] to minimize stigma or bias" since qualitative data would have provided important context to this statement. Similarly, the population's homogenous age of the listeners (95% between 18-23 years old) does raise concerns and the authors attribute potential influences ranging from generational differences in LGBTQ+ perception and the positivity effect that older listeners may perceive negative emotions differently.

Regarding conceptual framing, the paper adequately bridges perception literature and gender studies although more explicit connections within the discussion may enhance the overall paper. For example, Niedzielski (1999) showed that listeners hearing the same Detroit talker shifted their vowel judgments solely based on a social label ("Canadian" vs. "Detroit"), effectively altering what they believed they heard. This logic is consistent with the current study's pattern where perhaps knowledge of sexual orientation shifted perception of traits (e.g., sad, stuck-up) due to the social label, whereas broader attributions appear to depend on the acoustic signal indexed by perceived sexual orientation.

Overall, I found the paper to be thoughtfully provocative in evaluating associations between attributes and sexual orientation. While the statistics reported do partially support the aims by the authors, I am still hesitant about the participant sample. Undergraduates from a rural southeastern university potentially limit generalizability, so a more explicit and extensive statement of implications and planned next steps should be implemented. Perhaps providing more demographic information such as race or geographic origins could signal a more varied sample population. Regardless of the caveats, I do believe this study contributes to the literature due to label-manipulation of truthfulness and a well-controlled stimulus set. I recommend acceptance pending these revisions and copyediting tweaks (i.e. consistent capitalization of the conditions, attributes i.e. stuck up vs. stuck-up, and 'SO' vs. sexual orientation) and include emphasis on the need to have qualitative measures to contextualize qualitative details.

Line by Line Comments

[29] talkers -> talker
[38] listeners -> listeners'
[39] attributed -> attributes
[41] Delete "the"
[62] Delete duplicate "sound"
[91] attitudes -> attitude (Parallel number agreement with singular "emotion")
[94] selfish -> selfishness

[117] Add comma after “speech”
[124] Add “to” between “likely” and “indicate”
[125] Add “had” or “use” between “talkers” and “less”
[140] as the leader; -> as leaders;
[172] Third “talker” should be possessive as well “talkers’”
[213] Delete “a”
[248] Remove quotes around “boring” for consistency
[255 & 256] Add ‘’ around the number labels
[265] is -> are
[289] evidence -> evidenced
[301] that -> who
[301] analayses -> analyses
[320] stuck up -> stuck-up
[381] De-capitalize “Condition”
[334] stuck up -> stuck-up (in Table 1)
[411] capitalize second “referenced”
[434] capitalize “referenced”
[462] Result -> Results
[465-466] Plural agreement with noun “ratings” and verb “was” -> change ratings to rating or was to were for the sentence.
[490] Add “was” between “SO” and “referenced”
[493] trait -> traits
[500] was -> were
[510] Delete comma after “attributes”
[513] “coming off as” -> appearing
[536] listener’s -> listeners’
[553] expands -> expand
[630] over generalize -> overgeneralize
[634] don’t -> do not
[640] experiment -> experimental
[654] homogenous -> homogeneous
[662] LGTBQ+ -> LGBTQ+
[688] stuck up -> stuck-up
[688] talkers -> talker’s
[796] glmm TMB -> glmmTMB
[796] van Bentham KJ -> van Benthem KJ

References

Niedzielski, N. (1999). The effect of social information on the perception of sociolinguistic variables. *Journal of Language and Social Psychology*, *18*(1), 62–85. https://doi.org/10.1177/0261927X99018001005

Tripp, A., & Munson, B. (2023). Acknowledging language variation and its power: Keys to justice and equity in applied psycholinguistics. *Applied Psycholinguistics*, *44*(4), 495–513. https://doi.org/10.1017/S0142716423000206
