# Supplementary material for: Judgments of American English male talkers who are perceived to sound gay or straight: Which personal attributes are associated with each group of talkers?
Source: PLoS One. 2026 Apr 10;21(4):e0346897. doi: 10.1371/journal.pone.0346897 (PMC13068255; doi:10.1371/journal.pone.0346897)
Supplement: Attachment — Submitted filename: Response to Reviewers.docx [file pone.0346897.s002.docx]

We would like to thank the editor and the reviewers for their helpful suggestions and comments regarding our manuscript. The manuscript, we believe, improved with these insightful revisions. We have included most, if not all, of the indicated revisions, and the new revisions are in red font in the file “Revised Manuscript with Track Changes.” Below, we respond to each of the suggestions made by the editor and reviewers. We have also provided line numbers, so that the editor and reviewers can find these suggestions within the revised manuscript. We attempted to be as accurate as possible with the line numbers, so please excuse any mistakes.

Response to the Editor

1. We have uploaded seven files for further review: the rebuttal letter, the manuscript with track changes, the manuscript with no track changes, and the four figures. Please note that we believe that the figures should stand on their own; therefore, the figures use the term ‘sexual orientation’ instead of the SO acronym that was used in the manuscript. If the editor and/or reviewers would like us to change this, so that the figures contain the SO acronym, we are happy to oblige.

2. We have formatted the manuscript and figures in accordance with the information contained within the email that was sent to us on October 24.

Response to Reviewer #1

1. The Introduction needs to better contextualize the work within the multidisciplinary field.

The Introduction was extensively revised to meet this requirement. For instance, while we kept the discussion on perceiving sexual orientation (SO) [Lines 103-112] and attributes [Lines 113-160], we revised the section on SO and attributes [Lines 161-210]. This revised section includes a discussion on both stereotypes associated with gay and straight men [Lines 162 – 176] and discrimination in the workplace [Lines 193-210]. Additionally, we added a new section on the implications of knowing an individual’s SO [Lines 211-248]. This section includes a discussion on studies that did not reveal participants’ SO [Lines 212-221] and the emerging fields of social vision and social hearing [Lines 230-248].

2. Why were these attributes considered and not other attributes?

We further discussed the pilot study, which was used to identify the attributes in this manuscript. Briefly, we used those attributes that were identified most often by the listeners. The pilot study is discussed throughout the manuscript, and we hope that this discussion satisfies the reviewer’s question [Lines 91-92; 121-124; 323-342].

3. Why consider intelligence and confidence separately and not on a dimension?

These two attributes were both indicated by the listeners in the pilot study; the listeners differentiated between these two attributes. Here, we took a bottom-up approach and included those attributes most cited by participants in the pilot study. More information on the pilot study can be found throughout the manuscript [Lines 91-92; Lines 121-124; Lines 323-342].

4. Why not use attributes more stereotypically associated with gay and straight men?

We used the attributes that listeners indicated in the pilot study, and more information on the pilot study can be found throughout the manuscript [Lines 91-92; Lines 121-124; Lines 323-342]. We do agree with the reviewer that there is a necessity to further study these stereotypical attributes. To that end, we explained, in the Discussion, that future research studies should incorporate attributes stereotypically associated with gay and straight men [Lines 753-764].

5. Why not use neutral attributes?

As discussed previously, we used a bottom-up approach for this study by first asking listeners to identify those attributes most associated with various talkers. Unfortunately, neutral attributes were not indicated, at a high rate, by listeners; thus, we did not include them. However, in the Discussion, we do discuss further attributes that researchers could use in future studies. More information on the pilot study can be found throughout the manuscript [Lines 91-92; Lines 121-124; Lines 323-342], and more information on further attributes can be found throughout the Discussion [Lines 745-764].

6. The reviewer asked to incorporate the work of Blashill, Madon, and McAleer in the manuscript.

We found value in the work from these authors and included them within the manuscript. Blashill’s work is referenced in Line 166. Madon’s work is referenced in Lines 91, 170, 331, 747, and 764. McAleer’s work is referenced in Lines 68, 93, 129, 132, 139, and 750.

7. Why not include more social and emotion categories?

We used a bottom-up approach for this study, and we only included those attributes indicated most often by the listeners in the pilot study [Lines 91-92; Lines 121-124; Lines 323-342]. Furthermore, we did note that future studies should include more attributes, such as low valence attributes and high valence attributes [Lines 745-764].

8. Why are sadness and age more relevant than others?

Here, we were unclear what ‘others’ meant. For example, we were unclear if ‘others’ meant the other attributes investigated in this study or the other attributes (e.g., social and emotion) that the reviewer suggested? It was our intention to discuss each of the eight attributes equally. However, the rating results of the ‘sad’ attribute required more explanation, and we added this discussion in Lines 616-633.

9. The researchers haven’t considered the valence of the attributes.

Thank-you for the suggestion, and we did include a discussion on the valence of the attributes in both the Introduction [Lines 132-139; 745-752]. Additionally, we included a short discussion on another dimension: dominance [Lines 136-137; 749].

10. Terminology should be improved.

Thank-you, again, for the suggestion. We have strived to improve our terminology usage. For instance, we defined the terms attributes, attitudes, and emotions in the Introduction [Lines 114-120].

11. Possibly introduce the study as impression formation.

We agree with the reviewer and incorporated this term throughout the manuscript. For instance, it can first be found in Lines 61-69.

12. Justify why certain terms were used and why certain terms were not used.

We hoped to satisfy the reviewer’s suggestion with a greater description of the pilot study, as indicated above. Please refer to #2-5 and #7 above for a more detailed explanation.

13. Clarify why attributes are relevant to social consequences.

There is a relatively large section within the Introduction that details the social consequences of these attributes [Lines 177-210]. Here, we discussed stereotypes associated with gay and straight men, and the resulting consequences in the workplace and classroom. Additionally, there is a revised section in the Discussion that also discusses these social consequences [Lines 634-663].

14. Intelligence and confidence may matter in educational situations.

We have included Taylor and Raadt’s (2021) work on how stereotypical gay and straight male voices are perceived in an education setting [Lines 202-203; 214-216; 637-638].

15. Strengthen the rationale for testing the disclosure of the talkers’ SO.

Thank-you for this suggestion. We have included an additional section within the manuscript, ‘Knowledge of an individual’s self-identified SO’, to hopefully satisfy the reviewer’s concern [Lines 211-248]. Within this section, we discuss various auditory gaydar studies that did not disclose the talkers’ SO. Next, we discuss two studies in which knowledge of the talkers’ attributes changes how listeners perceive the talkers. Finally, we discuss both social vision and social hearing, and how these two fields provide motivation for our study.

16. The authors should consider the work on Gowen and Britt (2006) and Sulpizio et al. (2015) in terms of how the disclosure of a talker’s SO could influence the listener’s perception of them.

While we did include the work on Gowen and Britt within the paper [Lines 195-196; 647-648], we did not include the work of Sulpizio et al. (2015). However, we did include other references that we hope satisfies the reviewer’s concern. As mentioned above, we included a new section on how knowledge of an individual’s SO can affect a listener’s perception [Lines 211-248] and included within this section, we have included numerous references that hopefully strengthen our argument (e.g., Taylor & Raadt, 2021; Johnson et al., 2015).

17. The section on intersectionality needs more clarification. Why did Carnaghi et al. (2022) find that straight men were perceived as older?

We included more information about this study. For example, we included the statement that gay men were processed, by default, as young and that elderly men were processed, by default, as heterosexual [Lines 171-174].

18. Consider Sven Kachel’s work, as well as Lick and Johnson (2016) and Fasoli et al. (2023).

We did include one Kachel citation within the manuscript (Steffans, Niedlich, Kachel, & Methner, 2016) [Lines 166-169; 196-201; 660-662]. Here, we discussed Kachel’s work in terms of how a stereotype might affect one’s initial impression of another individual, and how gay and straight men might experience discrimination in the workplace. Furthermore, we included one work of Lick and Johnson within the manuscript (Johnson, Lick, & Carpinella, 2015) [Lines 232]; here we discussed their theory of social vision. Finally, we included the work of Fasoli et al. (2023) [Lines 78, 188, 198, and 216]; here we discussed how listeners place talkers in a social category and then associate various attributes with them. We also discussed how gay and straight men were perceived as equally competent and how LGBTQ+ individuals face discrimination within the workplace.

19. The pilot study needs to be explained in more detail.

We hoped to satisfy the reviewer’s comment by further discussing the pilot study. As outlined above, there is a new section within the Materials and Methods section that details the pilot study [Lines 325-344].

20. Present the study as exploratory since we are not putting forth direct hypotheses.

We agree with the reviewer in that we did not specifically predict which attributes would be associated with gay and straight talkers. Therefore, we presented the study as exploratory, and this is referenced throughout the manuscript [Lines 86, 100, 252, 574, 747].

21. Based on McAleer et al. (2014), we can offer possible directions while still keeping the study exploratory.

We included two works by McAleer: McAleer et al. (2014) [Lines 68, 132, 139, 752] and Baus et al. (2019) [Lines 93, 132,]. We discussed how listeners perceived personal attributes from talkers and the different dimensions of the social voice space.

22. Did we collect information on listener’s SO, age, and gender?

Yes, we did collect information about age and gender, but regrettably, did not collect information on SO [Lines 279-293]. We further discussed the age of our listeners both in the Methods and Materials section [Lines 279-293] and in the General Discussion [723-732], where we discussed some of the limitations of our sample. Within the manuscript, we did not report the listeners’ SO and, if memory serves correctly, there were few LGBTQ+ individuals within the listener sample. We also did not further discuss the listeners’ gender because, within the context of the manuscript, it seemed relatively less relevant, whereas age seemed more relevant and worthy of additional discussion.

23. Report the percentage of correct SO decisions, so that we know how SO was operationalized.

We discussed how the talkers were selected for the experiment [Lines 306-317]. We discussed the results of Tracy et al. (2015) in which each talker was rated by how confident listeners were of the talker’s SO. Next, we selected those talkers who fell at the extremes for inclusion in the present experiment. By extremes, we mean those self-identified gay talkers whom listeners labeled gay most often, those self-identified gay talkers whom listeners labeled straight most often, those self-identified straight talkers whom listeners labeled gay most often, and those self-identified straight talkers whom listeners labeled straight most often.

24. Please add a power analysis to justify the sample size and effect size for the analyses performed. Please elaborate on the effect sizes.

A sample size calculation for a poisson regression was completed using G*Power 3.1. Based on a predicted medium effect size, a beta of 0.8, and alpha of 0.05, the sample size of listeners needed for this experiment was calculated to be 164. However, the use of a nested data structure (i.e., stimuli nested within talkers nested within listeners) increases the standard error of a dataset. We estimated the interclass correlation between talkers and listeners to be approximately 0.1, which introduces approximately an 8-10% margin of error for the proposed sample of 24 talkers (Gelman & Hill, 2007); thus an additional ~15 participants were needed to account for this error. Our target sample size was thus ~180.

The recruitment method for this study was to offer course credit to undergraduate classes with a size ranging anywhere from 20-40 students. Based on the power analysis, we determined to offer this experiment to students in 9 classes (9 x 20 = 180) to ensure adequate power. Given that all students in each class were given the option to participate in the experiment, we expected this sampling method to result in our minimum recruitment target (i.e., 180), but anticipated that it may give us participants beyond this number.

The rate ratios (RR) provided throughout the manuscript were provided as measures of effect size as demonstrate the magnitude of the effect in a way that is directly comparable between effects and studies. Please let us know if you feel another effect size would be more appropriate.

Citation:

Gelman, A. & Hill, J. (2007). *Data Analysis Using Regression and Multilevel/Hierarchical Models.* Cambridge University Press.

25. Lack of control for regional accents is problematic as non-standard accents can affect first impressions.

We addressed the reviewer’s concern within the Materials and Methods section [Lines 295-305]. Talkers were required to be from the state of Ohio, but we did not control for regional accents within the state. However, the first author, who is a trained phonetician, did not notice any differences in regional accents of the talkers and, presumably, the listeners would not notice this either. Moreover, it was difficult to recruit gay talkers for the original study (e.g., Tracy et al., 2015), and those authors did not have the luxury of only sampling gay talkers who grew up in the Columbus, Ohio metropolitan area; therefore, the authors needed to expand their geographic region to the entire state of Ohio to recruit a large enough sample of gay talkers. We hope that these explanations satisfy the reviewer’s concerns.

26. Valence of the attributes should be considered as to whether it influences the attributions.

Within the Introduction, we included a more detailed section on the dimensions of attributes [Lines 132-139] and discussed where our chosen attributes fall within these dimensions. We admit that our attributes were not balanced along these dimensions. For example, we did not include equal numbers of high valence attributes and low valence attributes. We also did not include equal numbers of high dominance attributes and low dominance attributes. This is a shortcoming in the current exploratory study, and we discussed including additional attributes in future experiments within the General Discussion [Lines 747-754].

27. Mad and angry are different. Bring us back to definitions and different natures of attributes.

We took care to use consistent and clear definitions of both emotions and attitudes [Lines 114-120]. Furthermore, we also noted that the listeners in the pilot study described the talkers as mad (i.e., they did not use the term *angry*), and we wanted to utilize the terminology used by the listeners [Lines 126-128]. We do realize that there are different definitions of both mad and angry, as the reviewer notes. We conducted a second experiment to investigate whether listeners associated these attributes with one another, and we found that listeners, in fact, did associate these two terms with one another [Lines 538-572].

28. Were reaction times (RTs) recorded?

RTs were not recorded during the experiment. We feel that RTs are most informative when participants are given a two-alternative forced-choice task and always have their two pointer fingers over two buttons. In the current experiment, the participants chose among seven different options, which would involve finger movements from one button (e.g., ‘1’) to another (e.g., ‘7’). Even if participants decide, very quickly, that the talker rates a ‘7’, it might take time to move their finger from ‘1’ to ‘7’. Therefore, the RTs are recording, not their cognitive functions, but their finger movements. We did not feel that RTs would be informative in this experiment. A future experiment could ask participants if this talker is associated with a particular attribute and, instead of using a 7-point scale, the researchers could use a two-alternative forced-choice task (i.e., Yes or No).

29. I wonder whether it would be clearer to present the pairwise comparisons by keeping actual sexual orientation constant and varying the context, for instance, if a gay speaker is perceived as more stuck-up when truthfully described as gay, falsely described as straight, or when no information is provided. I think this would make it easier for the reader, though I appreciate the authors’ reporting of all comparisons.

We appreciate the reviewer’s suggestion; indeed, this is something we considered during our data analysis. However, the binary coding of the data for this study (e.g., talker either sounds gay or sounds straight) made us feel that contrasts that focused on either actual *or* perceived orientation were more appropriate. We thought an alternate study design which used categorical or continuous ratings of sexuality perception (e.g., “sounds very gay” to “sounds very straight”) would be a more interesting way to look at contrasts you are describing. This is an avenue we are considering for future work.

30. The section on anger attribution is unclear, as no tests of main effects or interactions are reported for anger. Without this, the results cannot be properly compared descriptively. It would also have been better to test mad and angry in the same sample for direct comparison.

The purpose of Experiment 1a was to determine whether participants were interpreting the word *mad* in Experiment 1 similarly to the word *angry*. We felt that having both words in the same experiment would unnecessarily bias participants to interpret the two words differently, and thus no direct comparison is available. Given the smaller sample size and purpose of Experiment 1a, we also did not include a manipulation of experimental condition (e.g., talker SO truthfully or falsely referenced). Thus, a comparison of the main effects between the two experiments would not be an apples-to-apples comparison.

We did, however, test the main effects of actual and perceived SO in Experiment 1a; neither main effect was statistically significant. This is likely due to the reduced sample size in Experiment 1 compared to Experiment 1, as we likewise found no significant main effects in all four traits examined in Experiment 1a (Stuck-Up, Intelligent, Boring). These results are now provided in the “AngerSOData” files in the Statistical Analyses folder within the OSF link (<https://osf.io/bc2wm/overview?view_only=8da8397267a04e21a34f1c367f626a9b>)

To add additional detail and further confirm the similar trends in results between “angry” and “mad”, we conducted Welch’s two-sample t-tests for the stratified talker groups and now present them in Table 4 [Lines 570-572] (replicated below).

|  |  | Mad | Angry | *t* (df) | *p* |
| --- | --- | --- | --- | --- | --- |
|  |  | Mean (SD) | Mean (SD) |  |  |
| Actual SO | Gay | 3.47 (1.89) | 3.49 (2.00) | 0.05 (29) | 0.962 |
|  | Straight | 3.48 (1.92) | 3.70 (1.95) | 0.53 (29) | 0.598 |
| Perceived SO | Gay | 3.86 (1.93) | 3.65 (1.94) | 0.51 (29) | 0.613 |
|  | Straight | 3.08 (1.80) | 3.53 (2.02) | 1.06 (29) | 0.296 |

31. There is not a link for the data repository.

Thank-you for the suggestion and this is something that we unfortunately did not include in the original manuscript. It is now included [Lines 416].

32. The conclusion should elaborate more on the significant interaction between condition/context and perceived SO.

We revised the Discussion section to, hopefully, satisfy the reviewer’s comment [Lines 590-617]. We tried to frame the results in terms of the social hearing model in which the speech signal itself activates the attributes regardless of the talkers’ label.

33. There needs to be more integration on impression formation and auditory gaydar.

As previously discussed, we revised the entire Introduction to better integrate these two concepts. For example, in the SO and attributes section, we started off by discussing attributes usually associated with both gay and straight men; here, we included the work of Clausell & Fiske (2005) [Lines 162-178]. Next, we transitioned to which attributes are associated with gay and straight male talkers and cited several relevant studies (e.g., Campbell-Kibler, 2011; Fasoli et al., 2017; Fasoli & Hegarty, 2020; Fasoli et al., 2023) [Lines 179-194].

34. Should the discussion on the acoustic correlates be included?

We briefly mentioned the acoustic correlates related to perceiving gay and straight speech [Lines 106-110] and the acoustic correlates related to perceiving emotions [Lines 132-147], attitudes [Lines 148-156], and age [Lines 157-160]. We eliminated the acoustic correlates section in the Discussion because, with the revision, it was irrelevant to the manuscript.

35. Expand the sadness results.

Thank-you for this suggestion and this was lacking in the original manuscript. There is a section in the Discussion that elaborates on the sadness results [Lines 618-635].

36. One limitation is that there is no consideration of female talkers and it’s important to investigate female talkers in future studies.

Yes, we agree with the reviewer and mentioned this comment within the manuscript [Lines 98-102].

37. Young people expect gay people to be more outgoing, and this can be linked to the work of Brambilla.

Unfortunately, we searched Brambilla’s work and could not find this mentioned. This is an unfortunate oversight on our part, and if the reviewer could suggest a citation, we would be more than willing to include it in a future version of the manuscript.

38. Within the Introduction, I would suggest clarifying the sentence to clarify that this is a belief.

We made the necessary change and included this wording [Line 73].

39. The references should be revised since some of them are not appropriate.

Thank-you for alerting us to this oversight. Upon revising the manuscript, we double-checked every citation to make sure that it was appropriate to that section of the manuscript. For instance, we agree with the reviewer that one of the Mack & Munson (2012) citations was not appropriate and it was removed.

Response to Reviewer #2

1. Be consistent on the use of SO and sexual orientation throughout the manuscript.

Thank-you for the comment and we have attended to this.

2. There are reservations about potentially underlying influences on listeners’ ements.

Thank-you for the suggestion. We have suggested that future studies could further investigate how individual differences among listeners could influence their judgments [Lines 742-744]. In other studies that we performed that were not mentioned within this manuscript, we did investigate how perceived contact with LGBTQ+ individuals might affect listeners’ judgments; however, this connection didn’t prove to be strong or informative. Another potential idea is to ask listeners to complete assessments concerning their thoughts and feelings toward LGBTQ+ people (e.g., Modern Homonegativity Scale). It may be the case that participants that rate high in homonegativity also rate talkers perceived as gay higher on attributes with a lower valence. While both reviewers offered many helpful suggestions on future research studies, we tried to limit these future studies for brevity. However, if the reviewer would like us to include a section on this particular measurement, we would be happy to do so.

3. Please include a description of Niedzielski (1999).

We have discussed Niedzielski (1999) in terms of how a geographic label affects how listeners perceive various vowels, as well as Drager (2006) in terms of how knowledge of a talker’s age affects how listener’s perceive various vowels [Lines 224-231].

4. Include a statement concerning the race and ethnicity of the listener population.

We included demographic data concerning our listener population [Lines 284-293]. Regrettably, we did not collect racial and ethnic information about our participants, but the student population at the University of North Carolina Pembroke (UNCP) is relatively diverse. We presumed that the listener population was similar to the larger student population, and indicated the demographic breakdown of the UNCP student population. Additionally, Tracy and Johnson (2015) found that listeners from both UNCP and the University of California Berkeley rated gay and straight talkers from Tracy et al. (2015) similarly. Thus, we feel confident that our results could be replicated with other listener populations.

Furthermore, within the Discussion section, we did revise the section on how a greater diversity in listeners’ ages might lead to further insights [Lines 703-722].

Citation:

Tracy, E.C., & Johnson, K. (2015, October). *Perceptual characteristics of gay-sounding and heterosexual-sounding speech*. Paper presented at the meeting of NWAV 44, Toronto, ON.

5. Be consistent with capitalization for the various conditions.

We have attended to this comment and are now consistent with capitalization.

6. Watch stuck-up versus stuck up.

We have attended to this comment and now consistent with stuck-up.

7. The reviewer also noted numerous small grammatical corrections to make within the manuscript.

We have attended to all these grammatical corrections and wish to thank the reviewer for their careful reading of the manuscript.
