# Supplementary material for: Judgments of American English male talkers who are perceived to sound gay or straight: Which personal attributes are associated with each group of talkers?
Source: PLoS One. 2026 Apr 10;21(4):e0346897. doi: 10.1371/journal.pone.0346897 (PMC13068255; doi:10.1371/journal.pone.0346897)
Supplement: Attachment — Submitted filename: Response_to_Reviewers_auresp_2.docx [file pone.0346897.s003.docx]

We would like to thank everyone for their insightful comments that helped us to improve our manuscript. The new revisions are in red font in the file “Revised Manuscript with Track Changes.” Below, we’ve responded to each of the suggestions made by the reviewers. Additionally, we’ve attempted to provide line numbers, so that the editor and reviewers can easily find the changes. We apologize if there are any discrepancies with the line numbers.

Response to Reviewer #1

1. Please be consistent with out-going versus outgoing.

We have changed all instances of “out-going” to “outgoing”; this is consistent throughout the manuscript.

2. Please be consistent with boring versus bored.

We have changed all instances of “bored” to “boring”; this is consistent throughout the manuscript.

3. The reviewer provided several small grammatical changes to make, and we have fixed all these small grammatical changes, as well as any other typos or grammatical changes we noticed during our read-through. Specifically, the reviewer noted they thought that [eo] was a typo. After checking this, we discovered that it was not a typo, but rather a missing reference. We have changed this to the correct reference [Line 178].

Response to Reviewer #2

1. Line 289, The participants mean age was 19.6 and this was not grammatical.

We assumed that the reviewer meant that we should include an apostrophe after "participants”, and we included this.

2. Line 351: 24 talkers X 2 utterances X 4 attributes (using "X" as a multiplication sign, which is non-standard in academic writing)

We deleted these sections (e.g., 24 talkers X 2 utterances X 4 attributes) in both Experiment 1 and Experiment 1a. Instead, both sections state the number of stimuli, trials, or lists [Lines 324, 332, 351, 361, 555].

3. The participants recruited for the experiment are relatively young (with a mean age of 19.6 years). Perceptual differences may exist among participants of different age groups and with varying social experience, and this limitation should be explicitly addressed in the Limitations section.

We agree that perceptual differences between age groups likely exist; we addressed differences in the social perception of LGBTQ+ communities in lines 708 – 728, as well as age-related differences in emotion perception in lines 729 – 738. In response to this comment, we further clarified that our listener sample is relatively young [Lines 707-708], and added information regarding other factors that may affect perceptual differences between age groups [Lines 736-738]

4. The data has not been made publicly available / Page is blank.

We apologize that the link was not working for the reviewer; it is possible that the link may have erroneously included an extra period at the end. All three authors independently verified that the supplied link is now working correctly. If you click on the link, it will take you to a page with two folders – Data and Statistical Analyses. Files are included in each of the two folders, even though the folders are indicated as 0 B. If you click on the Data folder, there are two publicly available data sets. If you click on the Statistical Analyses folder, there are four publicly available data files that contain all the statistical analyses for the current project.

5. The subheadings of the paper can be further refined to improve readability; Results Aim 1 changed to a concise description of Aim 1.

We changed the subheadings to align with the reviewer’s suggestion [Lines 419 – 420; 469].

6. Line 428: seen in Table 2 and Fig 1 – "Fig" should be spelled out in full as "Figure".

We changed all instances of the word “Fig” to the word to “Figure.”

7. The paper’s structure lacks clarity and appears disorganized; Experiment 1a includes a combined Results and Discussion section, while Experiment 1 only has a standalone Results section.

We deleted the “Results and Discussion” from Experiment 1a. It now reads “Results.” [Line 560]. As a result, the subheadings in Experiment 1 and Experiment 1a align more clearly with one another.

8. The sequencing of Experiment 1 and Experiment 1a is confusing and needs clarification.

To address this, we clarified earlier in the manuscript that anger and mad will be explored in Experiment 1a [Line 129]. Furthermore, we clarified the objectives of both Experiment 1 and Experiment 1a [Lines 252, 262, 270-271].

9. Lines 747–754: Although the study did not balance high- and low-valence attributes, the authors may still provide a relevant discussion of high- and low-valence attributes here.

We now include a discussion of the distribution of positive/negative valence in the attributes that were associated with gay and straight talkers and expand upon how a balanced design in future studies could further elucidate how listeners use features such as valence and dominance when making judgements of listener attributes [Lines 761-768],

10. Discussion on Sad Attribute; Conclusion of paragraph needs to be succinct; Summarize the key findings as to why sad demonstrated a different pattern.

We summarized the discussion on “sad” by highlighting the simplest, and perhaps, most likely explanation [Lines 634-637].
